# Injuries in Field Hockey Players: A Systematic Review

**DOI:** 10.1007/s40279-017-0839-3

**Published:** 2018-01-03

**Authors:** Saulo Delfino Barboza, Corey Joseph, Joske Nauta, Willem van Mechelen, Evert Verhagen

**Affiliations:** 1 Amsterdam Collaboration on Health and Safety in Sports, Department of Public and Occupational Health, Amsterdam Public Health Research Institute, VU University Medical Center, Van der Boechorststraat 7, 1081 BT Amsterdam, The Netherlands; 2 Australian Collaboration for Research into Injury in Sport and its Prevention, Federation University Australia, Lydird Street South, Ballarat, VIC 3350 Australia; 3 School of Human Movement and Nutrition Sciences, Faculty of Health and Behavioural Sciences, University of Queensland, Brisbane, QLD 4072 Australia; 4 Division of Exercise Science and Sports Medicine, Department of Human Biology, Faculty of Health Sciences, University of Cape Town, Anzio Road, Observatory 7925, Cape Town, South Africa; 5 School of Public Health, Physiotherapy and Population Sciences, University College Dublin, Belfield, Dublin 4, Ireland

## Abstract

**Background:**

To commence injury prevention efforts, it is necessary to understand the magnitude of the injury problem. No systematic reviews have yet investigated the extent of injuries in field hockey, despite the popularity of the sport worldwide.

**Objective:**

Our objective was to describe the rate and severity of injuries in field hockey and investigate their characteristics.

**Methods:**

We conducted electronic searches in PubMed, Embase, SPORTDiscus, and CINAHL. Prospective cohort studies were included if they were published in English in a peer-reviewed journal and observed all possible injuries sustained by field hockey players during the period of the study.

**Results:**

The risk of bias score of the 22 studies included ranged from three to nine of a possible ten. In total, 12 studies (55%) reported injuries normalized by field hockey exposure. Injury rates ranged from 0.1 injuries (in school-aged players) to 90.9 injuries (in Africa Cup of Nations) per 1000 player-hours and from one injury (in high-school women) to 70 injuries (in under-21 age women) per 1000 player-sessions. Studies used different classifications for injury severity, but—within studies—injuries were included mostly in the less severe category. The lower limbs were most affected, and contusions/hematomas and abrasions were common types of injury. Contact injuries are common, but non-contact injuries are also a cause for concern.

**Conclusions:**

Considerable heterogeneity meant it was not possible to draw conclusive findings on the extent of the rate and severity of injuries. Establishing the extent of sports injury is considered the first step towards prevention, so there is a need for a consensus on injury surveillance in field hockey.

**Electronic supplementary material:**

The online version of this article (10.1007/s40279-017-0839-3) contains supplementary material, which is available to authorized users.

## Key Points

**Table:** 

Substantial heterogeneity between studies prevents conclusive findings on the extent of the rate and severity of injuries in field hockey.
Injury prevention efforts in field hockey may benefit from a consensus on the methodology of injury surveillance.

## Introduction

Field hockey is an Olympic sport played by men and women at both recreational and professional levels. The five continental and 132 national associations that are members of the International Hockey Federation [1] demonstrate the high level of popularity of field hockey worldwide. Field hockey participation may contribute to players’ health through the well-known benefits of regular exercise. However, participation in field hockey also entails a risk of injury [2].

In general, sports injuries result in individual and societal costs [3], hamper performance, and compromise a teams’ success over the sporting season [4, 5]. Therefore, injury prevention strategies are of great importance for teams at both recreational and professional levels. Establishing the extent of the injury problem is considered the first step towards effective prevention [6]. In field hockey, as well as in other sports, this information can aid researchers and health professionals in developing appropriate strategies to reduce and control injuries [6].

To the best of our knowledge, no systematic reviews have provided a synthesis of information on injuries sustained by field hockey players. Systematic reviews involve gathering evidence from different sources to enable a synthesis of what is currently known about a specific topic (e.g., injuries) and may facilitate the link between research evidence and optimal strategies for healthcare [7]. Therefore, the aim of this study was to systematically review the literature on injuries sustained by field hockey players, in order to describe the extent of such injuries in terms of rate and severity as well as to identify injury characteristics according to body location, type, and mechanism of injury.

## Methods

### Information Sources and Search Strategy

Electronic searches were conducted in PubMed, Exerpta Medical Database (Embase), SPORTDiscus, and Cumulative Index to Nursing and Allied Health Literature (CINAHL) databases with no limits on the publication date. The search strategy combined keywords for injury, field hockey, and study design: ((((((((((((injur*) OR traum*) OR risk*) OR overuse) OR overload) OR acute) OR odds) OR incidence) OR prevalence) OR hazard)) AND (((field AND hockey)) OR (hockey NOT ice))) AND (((prosp*) OR retrosp*) OR case*). The detailed search strategy for each database can be found Appendix S1 in the Electronic Supplementary Material (ESM). The last search was conducted on 31 May 2017.

### Eligibility Criteria

Studies were eligible for inclusion if they were published in the English language in a peer-reviewed academic journal, were prospective cohort studies, and observed all possible injuries sustained by field hockey players during the period of the study (i.e., studies that looked only at specific injuries were not included). To minimize the possibility of recall bias, only prospective cohort studies were included [8, 9]. Studies were not included if they described field hockey injuries together with those from other sports, and specific data on field hockey could not be distinguished. Conference abstracts were not included.

### Study Selection and Data Collection Process

Two reviewers (SDB and CJ) independently screened all records identified in the search strategy in two steps: title and abstract screening, and full-text screening. References of full texts were also screened for possible additional studies not identified in the four databases. Conflicts between reviewers’ decisions were resolved through discussion. A third reviewer (EV) was consulted for consensus rating when needed.

One reviewer (SDB) extracted the following information from the included studies: first author, publication year, country in which the study was conducted, primary objective, setting, follow-up period, number and description of field hockey players, injury definition, injury data collection procedure, number of injured players, number of injuries sustained by players during the study, and severity of injuries (Table 1). The number of injuries normalized by exposure to field hockey (i.e., injury rate) was also extracted. In addition, information on injury according to body location, type of injury, mechanism, and player position was gathered whenever possible. When different studies used the same dataset (Table 1), the results of such studies were combined in one row in all other tables for simplicity.

**Table 1:** Characteristics of prospective studies on field hockey injuries arranged by year of publication (least recent to most recent)

Study (country)	Primary objective	Setting and follow-up period	Description of field hockey players	Injury definition (summary)	Injury data collection	Injured players	Number of injuries	Severity of injury
Weightman and Browne 1975 (UK) [11]	Survey injuries in 11 selected sports	Season (8 months)	Men (25 clubs) and women (36 clubs). Number, age, and level NR	NR	Sport clubs’ secretaries form	NR	117	Average TL. Women: 1.5 days; Men: 6.5 days
Clarke and Buckley 1980 (USA) [13]	Preliminary overview of injury experiences among collegiate women athletes reported to the National Athletic Injury/Illness Reporting System during its first 3 operational years	Season (3 years)	High-school women from annual average of 16 teams. Number and age NR	An injury causing the athlete to miss at least 1 week of participation (≥ 1 week TL)	Athletic trainer injury report form	NR	NR	TL and consequences: > 3 weeks: 23%; Surgery: 5%
Zaricznyj et al. 1980 (USA) [12]	Analyze causes and severity of sports injuries in a total school-aged population	School season (1 year)	65^a^ school-aged players (5–17 years)^b^. Number and sex NR	Any traumatic act against the body sufficiently serious to have required first aid, school and insurance accident reports, or medical treatment (MA)	Principals, coaches, sport supervisors, ERs, school insurance company, local physician’s injury form	NR	25	Injury type and consequences (NR)
Mathur et al. 1981 (Nigeria) [15]	Determine sites and types of common injuries associated with competitive sports popular in Nigeria	Season (8 weeks)	212 players. Sex, level, and age NR	NR	Athlete self-report questionnaire	NR	641	NR
Rose 1981 (USA) [14]	Describe women’s field hockey injuries at the California State University in Long Beach	Season (4 years)	University women. Number and age NR	Minor injury: required MA of team physician in some cases but handled mainly by the trainer and produced no or limited disability. Major injury: required MA of team physician and produced definite disability needing follow-up care (medical/trainer attention)	NR	NR	81	Injury type and consequences. Minor: 82.7%^c^; Major: 17.3%^c^
Martin et al. 1987 (USA) [16]	Detail injury experiences of 1985 Junior Olympics	1985 Junior Olympic games (7 days)	53 women. Age NR	Injuries severe enough to withhold athlete from competition, at least temporarily, and to require formal medical evaluation by the trainer (medical/trainer attention and TL)	Medical staff report form	15.1% (8)	9	Tissue damage. Outcome NR
Jamison and Lee 1989 (Australia) [18]	Compare injuries during Australian Women’s Hockey Championships, 1984 (on grass) and 1985 (on Astroturf)	Championship (2 years)	110 women playing at Australians’ state teams. Age NR.	NR	Athletes self-report questionnaires	NR	178	NR
McLain and Reynolds 1989 (USA) [17]	Investigate sports injuries at a large high-school	School season (7 months)	46 high-school women. Age NR	Any incident resulting from athletic participation that keeps athletes from completing a practice or game or causes athlete to miss a subsequent practice or game (TL)	Athletic trainer injury evaluation sheet	6% (3)	NR	Average TL: 3.3 days
Fuller 1990 (Country NR) [19]	Study whether a characteristic pattern of injuries and their causation existed at county and territorial competition levels in women’s field hockey on synthetic turf pitches	Competitive season (2 years)	Women. Number, level, and age NR	Presence of pain, discomfort, or disability arising during or as consequence of playing in a hockey match and for which physiotherapy treatment, advice, or handling was given (MA)	Researcher observation and contact with athletes	NR	135	TL. ≤ 2 days: 90%; > 2 days: 10%
Cunningham and Cunningham 1996 (Australia) [20]	Obtain data relating to frequency, type, mechanism, severity of sports injuries incurred during or related to competition	1994 Australian University Games (6 days)	466^c^ university players, aged 17–47 years. Sex NR^b^	Any incident during warm-up or competition that required MA, on-field management to enable continued participation, or removal from the playing field (MA)	Attending officer injury surveillance form	33.5% (156)	181	Required treatment and injury outcome (NR)
Fawkner et al. 1999 (Australia) [22]	Examine relationship between hassles and athletic injury	Season (13 weeks)	26 professional women aged 26 years on average^b^	Medical problem resulting from either participation in training or competition, required MA, and restricted further participation in either training or a competition for at least 1 day post occurrence (MA and ≥ 1 day TL)	Coach recording form	23% (6)	NR	NR
Powell and Barber-Foss 1999^d^ (USA) [21]	Describe injury patterns in ten high school sports	Season (2 years)	High-school women, number, age NR	(1) injury causing cessation of participation in current game or practice and prevented player’s return to that session, (2) injury causing cessation of a player’s customary participation on the day following the day of onset, (3) any fracture, even though athlete did not miss any regularly scheduled session, (4) any dental injury, including fillings, luxations, and fractures, and (5) any mild brain injury requiring cessation of player’s participation for observation before returning, either in current or next session (MA or ≥ 1 day TL)	Athletic trainer injury form	(445)	510	TL. < 8 days: 79.6%; 8–21 days: 13.3%; > 21 days: 7.1%
Stevenson et al. 2000 (Australia) [23]	Describe trends in recreational sports injury in Perth, Western Australia	Winter season (5 months)	393 non-professional men (170) and women (223) aged 25 years on average	Injury occurring while participating in sport and leading to one of the following consequences: reduction in amount or level of sports activity, need for advice or treatment, and/or adverse economic or social effects (TL or MA and/or adverse economic/social effects)	Assisted telephone interviewing with athletes	28% (198)	279	Injury treatment (NR)
Finch et al. 2002^e^ (Australia) [24]	Describe incidence of injury over two consecutive sporting seasons in a prospective cohort of community-level sporting participants within Australian football, hockey, basketball, netball	Two consecutive winter seasons (10 months)	280 non-professional men (116)^c^ and women (164)^c^ aged 25 years on average	One that occurred while participating in sport and that led to reduction in the amount or level of sport activity and/or need for advice or treatment and/or adverse economic or social effects (TL or MA and/or adverse economic/social effects)	Assisted telephone interviewing with athletes	31% (87)	445	Injury treatment (NR)
Junge et al. 2006 (Greece) [25]	Analyze and compare incidence, characteristics, and causes of injuries in all team sport tournaments during 2004 Olympic Games	2004 Olympic Games (19 days)	Olympic men and women. Number and age NR	Any physical complaint incurred during the match that received MA from the team physician, regardless of the consequences with respect to absence from the match or training (MA)	Physician injury report form	NR	44	Estimated TL. None: 50%^c^; 1–3 days: 27.3%^c^; 4–7 days: 9.1%^c^; > 1 month: 2.3%^c^; Unspecified: 2.3%^c^; Missing: 9.1%^c^
Dick et al. 2007^f^ (USA) [28]	Review 15 years of NCAA injury surveillance data for women’s field hockey	Season (15 years)	5385 high-school women. Age NR	One that (1) occurred due to participation in an organized intercollegiate practice or competition and (2) required MA by a team-certified athletic trainer or physician and (3) resulted in restriction of the student athlete’s participation or performance for 1 or more calendar days beyond the day of injury (MA and ≥ 1 day TL)	Athletic trainer injury report form	NR	3286	> 10 TL days. Game injuries: 15%; Practice injuries: 13%
Hootman et al. 2007^f^ (USA) [26]	Summarize 16 years of NCAA injury surveillance data for 15 sports	Season (15 years for field hockey)	5385 high-school women. Age NR	One that (1) occurred as a result of participation in an organized intercollegiate practice or competition and (2) required MA by team-certified athletic trainer or physician and (3) resulted in restriction of the student athlete’s participation or performance for ≥ 1 calendar days beyond the day of injury (MA and ≥ 1 day TL)	Athletic trainer injury report form	NR	3286	> 10 TL days. Game injuries: 15%; Practice injuries: 13%
Rauh et al. 2007^d^ (USA) [27]	Determine patterns of new and subsequent injuries among female athletes participating in interscholastic sport	Season (2 years)	High-school women. Number and age NR	(1) Any injury causing cessation of participation in current game or practice and prevented player’s return to that session; (2) any injury causing cessation of player’s customary participation on the day following the day of onset; (3) any fracture, even though the athlete did not miss any regularly scheduled session; (4) any dental injury, including fillings, luxations, and fractures, (5) any mild brain injury requiring cessation of player’s participation for observation before returning, either in the current or next session (MA or ≥ 1 day TL)	Athletic trainer injury form	(445)	510	TL. < 8 days: 79.6%; 8–21 days: 13.3%; > 21 days: 7.1%
Junge et al. 2009 (China) [30]	Analyze frequency, characteristics, and causes of injuries incurred in competitions and/or training during 2008 Olympic Games	2008 Olympic Games (16 days)	382 Olympic men and women aged 26 years on average^b^	Any musculoskeletal complaint newly incurred due to competition and/or training during the XXIXth Olympiad in Beijing that received MA regardless of consequences with respect to absence from competition or training (MA)	Physician injury report form	20.4% (78)	78	Estimated TL: 3.5% of players
Rishiraj et al. 2009 (Canada) [29]	Identify rates, profiles, and severity of injuries associated with participating in under-21 age representative field hockey team	Season (5 years)	75 women aged 18 years on average representing BC Women’s Field Hockey Federation	Any event during team or team-related game, practice, or activity (on or off the playing surface) requiring attention by team’s therapist or physician and subsequent game/practice TL (MA and ≥ 1 day TL)	Athletic therapist injury reporting system	NR	198	TL. < 7 days: 81%; 8–12 days: 17%; > 21 days: 2%
Engebretsen et al. 2013 (UK) [31]	Analyze injuries and illnesses during 2012 Olympic Games	2012 Olympic games (19 days)	388 Olympic men (196) and women (192). Age NR	New or recurring musculoskeletal complaints or concussions (injuries) incurred during competition or training during London Olympic Games receiving MA, regardless of consequences regarding absence from competition or training (MA)	Physician injury report form	17% (66)	66	TL. ≥ 1 day: 37.9%; ≥ 7 days: 15.2%
Theilen et al. 2016 [multiple countries (Table 3)] [32]	Investigate incidence and severity of injuries during international field hockey tournaments in 2013	16 International Hockey Federation tournaments^g^	Professional men and women. Number and age NR	A new musculoskeletal symptom or concussion that led to time stoppage when player was unable to continue playing during competition (TL)	Medical officer injury form	NR	236^c^	NR

*BC* British Columbia, *MA* medical attention, *ERs* Emergency rooms, *NATA* National Athletic Trainers’ Association, *NCAA* National Collegiate Athletic Association, *NR* not reported, *TL* time loss

^a^Players participating in school teams. Does not include physical education, non-organized, and community practice (that are reported in the study)

^b^Data from the whole cohort (not only from field hockey players)

^c^Calculated from presented data

^d^Studies using the same data from 1995–1997 NATA High School Injury database

^e^Finch et al. [24] is a follow-up study of Stevenson et al. [23]

^f^Studies using the same data from 1988–2003 NCAA Injury Surveillance System

^g^Tournament durations in 2013 ranged from 3 to 10 days. The specific duration of each tournament can be found at https://tms.fih.ch/fih/home/

### Risk of Bias Assessment

Two independent reviewers (SDB and CJ) assessed the risk of bias in the included studies using ten criteria previously used in systematic reviews on sports injury [9, 10]. All criteria were rated as 1 (i.e., low risk of bias) or 0 (i.e., high risk of bias). When insufficient information was presented in a study to rate a specific criterion as 1 or 0, the rating was categorized as ‘unable to determine’ (UD) and counted as 0. The assessment of each reviewer was compared, and conflicts were resolved through discussion. The ten criteria are described in Table 2.

**Table 2:** Risk-of-bias assessment of studies on field hockey injuries according to ten criteria

Study	Criteria										Score
	1	2	3	4	5	6	7	8	9	10	
Weightman and Browne 1975 [11]	0	1	0	0	1	0	1	0	1	1	5
Clarke and Buckley 1980 [13]	1	1	1	0	UD	1	1	1	1	1	8
Zaricznyj et al. 1980 [12]	1	1	1	1	1	0	0	0	1	1	7
Mathur et al. 1981 [15]	0	1	0	0	UD	1	1	0	0	0	3
Rose 1981 [14]	1	1	1	0	UD	UD	UD	0	1	0	4
Martin et al. 1987 [16]	1	1	1	1	1	1	1	1	0	0	8
Jamison and Lee 1989 [18]	0	1	1	0	1	1	1	0	1	0	6
McLain and Reynolds 1989 [17]	1	1	1	0	1	1	1	1	1	0	8
Fuller 1990 [19]	1	1	0	0	UD	1	1	0	1	0	5
Cunningham and Cunningham 1996 [20]	1	1	1	1	UD	1	1	0	0	0	6
Fawkner et al. 1999 [22]	1	1	1	0	1	0	0	0	0	0	4
Powell and Barber-Foss 1999 [21]	1	1	1	0	UD	1	0	1	1	1	7
Stevenson et al. 2000 [23]	1	1	1	1	0	1	1	0	0	1	7
Finch et al. 2002 [24]	1	1	1	1	0	1	1	0	1	1	8
Junge et al. 2006 [25]	1	1	1	1	0	1	1	1	0	1	8
Dick et al. 2007 [28]	1	1	1	0	UD	1	1	1	1	1	8
Hootman et al. 2007 [26]	1	1	1	0	UD	1	1	1	1	1	8
Rauh et al. 2007 [27]	1	1	1	0	UD	1	0	1	1	1	7
Junge et al. 2009 [30]	1	1	1	1	1	1	1	1	0	0	8
Rishiraj et al. 2009 [29]	1	1	1	0	UD	1	1	1	1	1	8
Engebretsen et al. 2013 [31]	1	1	1	1	1	1	1	1	0	0	8
Theilen et al. 2016 [32]	1	1	1	1	1	1	1	1	0	1	9
**Total,** *** n*** **(%) of studies**	**19 (86)**	**22 (100)**	**19 (86)**	**9 (41)**	**9 (41)**	**18 (82)**	**17 (77)**	**12 (55)**	**13 (59)**	**12 (55)**	

Risk of bias: low = 1, high = 0. Unable to determine fields (UD) were counted as zero in the score

**1** definition of injury clearly described;** 2** prospective design that presents incidence or prevalence data; **3** description of field hockey players (e.g., recreational or professional level); **4** the process of inclusion of athletes in the study was at random (i.e., not by convenience) or the data collection was performed with the entire target population; **5** data analysis performed with at least 80% of the athletes included in the study; **6** injury data reported by players or by a healthcare professional; **7** same mode of injury data collection used; **8** injury diagnosis conducted by medical professional; **9** follow-up period of at least 6 months; **10** incidence or prevalence rates of injury expressed by a ratio that represents both the number of injuries as well as the exposure to field hockey (i.e., number of injuries/hours of field hockey exposure, or number of injuries/sessions of field hockey exposure)

## Results

### Search Results

**Fig. 1:**
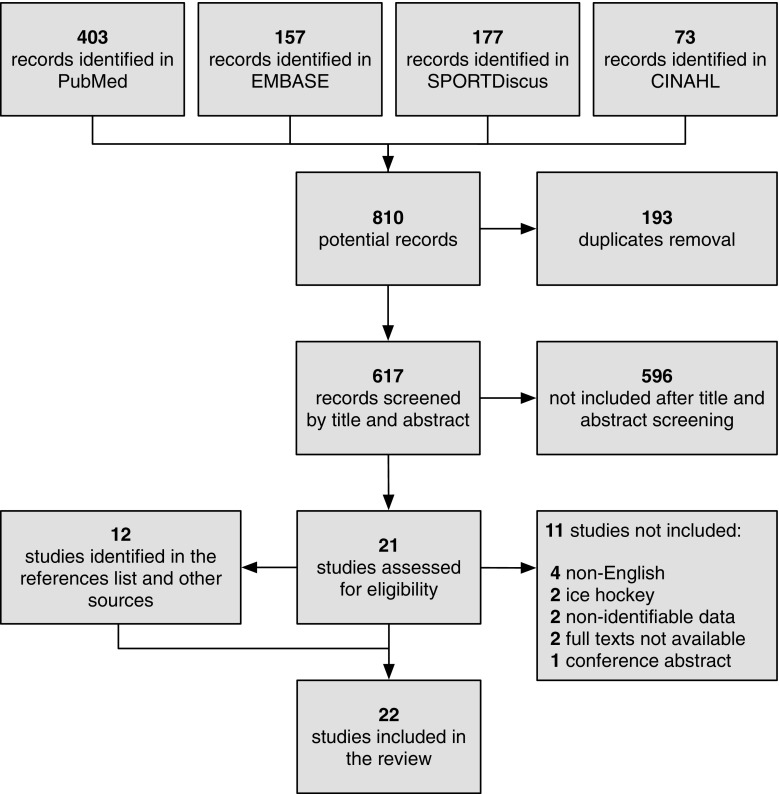
Flowchart of the studies during the inclusion process. Electronic searches were conducted in PubMed, Exerpta Medical Database (Embase), SPORTDiscus, and Cumulative Index to Nursing and Allied Health Literature (CINAHL) databases with no limits on the publication date

We retrieved 810 records from the four databases. Of those, 193 were duplicates. After screening 617 titles and abstracts and 21 full texts, ten studies matched the inclusion criteria. Screening the references of the full texts resulted in 12 additional records. In the end, 22 studies were included in the review. The flowchart of the inclusion process is presented in Fig. 1.

### Description of the Included Studies

The characteristics of the 22 studies included in this review are presented in Table 1. Studies included in this review were published between 1975 and 2016, with 12 (55%) published before 2000 [11–22] and ten (45%) published from 2000 onwards [23–32]. Two studies used the same dataset from the National Athletic Trainers’ Association (NATA) High School Injury database [21, 27], and two used the same dataset from the National Collegiate Athletic Association (NCAA) Injury Surveillance System [26, 28]. One study [24] was the follow-up of a previous study [23].

Six studies (27%) focused on describing field hockey injuries only [14, 18, 19, 28, 29, 32]. The other 16 studies (73%) described the epidemiology of injuries in field hockey together with those in other sports [11–13, 15–17, 20–27, 30, 31]. The period of follow-up varied between studies from a 6-day championship tournament [20] to 15 consecutive seasons of field hockey [28]. The sample size varied between 26 [22] and 5385 participants [28]. However, seven studies (32%) did not report the number of field hockey players studied [11, 13, 14, 19, 21, 25, 27].

The definition of injury varied across the studies. Common criteria to define an injury as recordable were a musculoskeletal condition requiring medical attention and/or leading to field hockey time loss (Table 1). The proportion (%) of injured players varied from 6% (in 7 months of high school) to 33% (in 6 days of university games). Twelve studies (55%) did not report the number or proportion of players who had sustained an injury over the study period [11–15, 18, 19, 25, 26, 28, 29, 32].

### Risk-of-Bias Assessment

Table 2 shows the risk-of-bias assessment for the 22 included studies. The total score ranged from three to nine of a possible ten points. The studies published during and since 2000 scored higher (range 7–9) [23–31]. Three studies (14%) did not provide a clear definition of injury [11, 15, 18], and three did not describe any characteristics of the players studied [11, 15, 19]. These studies were published before the year 2000.

Nine studies (41%) included a random sample of players or studied the entire target population [12, 16, 20, 23–25, 30–32]. Eighteen studies (82%) collected injury data directly from players or medical professionals, 17 studies (77%) used only one method (i.e., not multiple methods) to collect injury data during the study [11, 13, 15–20, 23–26, 28–32], and one study (5%) did not describe the data collection procedure at all [14].

Twelve studies (55%) employed a medical professional to diagnose injuries [13, 16, 17, 21, 25–32]. The follow-up period of 13 studies (59%) was over 6 months [11–14, 17–19, 21, 24, 26–29], and 12 studies (55%) expressed ratios that represented both the number of injuries and the exposure to field hockey [11–13, 21, 23–29, 32].

### Injury Extent in Field Hockey

#### Injury Rates

In total, 12 studies (55%) reported the number of injuries normalized by player exposure (i.e., injury rate). The injury rates reported in each of these studies are presented in Table 3, and were divided into two categories: (1) number of injuries per 1000 player-hours of field hockey exposure (i.e., time at risk) [11, 12, 23–25, 32] and (2) number of injuries per 1000 player-sessions (i.e., sessions at risk) [13, 21, 25–29]. One study reported the number of injuries according to both player-hours and player-sessions at risk [25].

**Table 3:** Number of field hockey injuries (and 95% confidence intervals) per 1000 player-hours and player-sessions arranged by players’ characteristics

Study	Players’ characteristics	Setting	Injury definition summary	Players’ exposure (hours)	Number of injuries per 1000 player-hours	Players’ exposure (sessions)	Number of injuries per 1000 player-sessions
Weightman and Browne 1975 [11]	Unspecified	Season	NR	122,074^a^	1.0 (0.8–1.1)^a^		
	Men			70,874^a^	1.0 (0.8–1.3)^a^		
	Women			51,200^a^	1.3 (0.9–1.6)^a^		
Zaricznyj et al. 1980 [12]	School players aged 5–17 years, sex NR	Season	MA	14,286^a^	0.1 (0.0–1.4)^a^		
Clarke and Buckley 1980 [13]	High-school women, age NR	Season	≥ 1 week TL				1.0^b^
Powell and Barber-Foss 1999^c^ [21]	High-school women, age NR	Season	MA or ≥ 1 day TL			138,073	3.7 (3.4–4.0)^a^
		Game				66,122^a^	4.9 (4.4–5.4)^a^
		Practice				58,125^a^	3.2 (2.7–3.7)^a^
Dick et al. 2007^d^ [28]	High-school women, age NR	Season	MA and ≥ 1 day TL			716,910^a^	4.6 (4.4–4.7)^a^
		Game				155,370^a^	7.9 (7.4–8.3)
		Practice				561,540^a^	3.7 (3.5–3.9)
Rishiraj et al. 2009 [29]	Under-21 aged women from the British Columbia Women’s Field Hockey Federation	Season	MA and ≥ 1 day TL			2828	70.0 (30.2–79.8)^a^
		Game				578	67.5 (45.6–89.3)^a^
		Practice				2250	68.0 (57.1–78.9)^a^
Finch et al. 2002^e^ [24]	Non-professional men and women, average age 25 years	Winter season	TL or MA and/or adverse economic/social effects	29,276^a^	15.2 (13.8–16.7)		
Junge et al. 2006 [25]	Olympic players, age NR	2004 Olympic Games	MA	1322^a^	33 (23–43)^a^	1133^a^	39 (27–50)
	Men			770	47 (32–62)	660	55 (37–72)
	Women			552	14 (4–24)	473	17 (5–29)
Theilen et al. 2016 [32]	Professional players, age NR	2013 FIH tournaments	TL	6519^a^	36.2 (31.6–40.8)^a^		
	Men			4825	48.3 (30.9–68.8)		
		Africa Cup of Nations (Kenya)		154	90.9 (38.4–143.4)^a^		
		East Asia Games (China)		154	90.9 (38.4–143.4)^a^		
		Junior World Cup (India)		1129	27.4 (17.4–37.5)^a^		
		Oceania Cup (NZ)		154	77.9 (28.4–127.4)^a^		
		Sultan of Johor Cup (Malaysia)		462	28.1 (11.1–45.1)^a^		
		World League Round 2 (India)		385	44.2 (21.5–66.9)^a^		
		World League Round 2 (Russia)		385	44.2 (21.5–66.9)^a^		
		World League Round 2 (France)		385	26.0 (7.4–44.6)^a^		
		World League Round 2 (Brazil)		385	20.8 (3.4–38.2)^a^		
		World League Semi-final (Malaysia)		616	42.2 (25.2–59.3)^a^		
		World League Semi-final (The Netherlands)		616	39.0 (22.5–55.4)^a^		
	Women			1694	29.1 (18.6–39.7)		
		4 Nations Tournament (NZ)		154	26.0 (0.0–67.3)^a^		
		East Asia Games (China)		154	26.0 (0.0–67.3)^a^		
		World League Final (Argentina)		616	26.0 (12.1–39.8)^a^		
		World League Round 2 (India)		385	44.2 (21.5–66.9)^a^		
		World League Round 2 (Brazil)		385	23.4 (5.4–41.4)^a^		

*FIH* International Hockey Federation, *MA* medical attention, *NR* not reported, *NZ* New Zealand, *TL* time loss

^a^Calculated according to presented data

^b^Impossible to calculate 95% confidence interval

^c^Same data as Rauh et al. 2007 [27]

^d^Same data as Hootman et al. 2007 [26]

^e^A follow-up study of Stevenson et al. 2000 [23]

In the studies describing injuries according to players’ time at risk, injury rates ranged from 0.1 injuries (in school-aged players) [12] to 90.9 injuries (in Africa Cup of Nations) [32] per 1000 player-hours of field hockey (Table 3). The injury rate in the studies describing injuries according to players’ sessions at risk varied from one injury (in high-school women) [13] to 70 injuries (in under-21 age women) [29] per 1000 player-sessions. The injury rates were higher in games than in training sessions in two [21, 28] of the three studies that investigated this outcome [21, 28, 29]. In major tournaments, injury rates were higher in men [25, 32].

#### Injury Severity

Table 1 presents the classification of injuries according to severity. Most of the studies (55%) used field hockey time loss to report the severity of injuries [11, 13, 17, 19, 21, 25–31], but reported the days of time loss differently. Some studies reported the average days of time loss [11, 17] and others used diverse cut-off points to report injury-related days of time loss, such as two days [19], eight days [21, 27], and ten days [26, 28]. The majority of injuries were in the less severe category in all studies reporting days of time loss due to injury, regardless of the cut-off points used [13, 14, 19, 21, 25, 28, 29, 31]. Six studies (27%) included severity measures in the methodology but did not specify the number or proportion of injuries according to severity in the results [12, 16, 20, 23, 24, 32]. Three studies (14%) did not mention severity of injury at all [15, 18, 22].

### Injury Characteristics in Field Hockey

#### Body Location and Types of Injury

Fifteen studies (68%) described injuries according to the affected body location [12–16, 18, 19, 21, 24, 25, 27–29, 31, 32]. Table 4 presents the proportion (%) of injuries according to body location reported in these studies. The most common site of injury was the lower limbs (ranging from 13% [25] to 77% [18] of all injuries), followed by head (2% [13] to 50% [25]), upper limbs (0% [16] to 44% [12]), and trunk (0% [18] to 16% [28]). In the lower limbs, injuries were more frequent in the knee, ankle, lower leg, and thigh (Table 4).

**Table 4:** Proportion (%) of field hockey injuries by body location

Study	Head, neck, face	Upper limbs					Trunk, upper and lower back	Lower limbs							Other, unspecified
		Hand, finger, wrist	Upper arm, forearm	Elbow	Shoulder	Total upper limbs		Ankle	Foot, toes	Lower leg	Thigh	Knee	Hip, groin, pelvis	Total lower limbs	
Clarke and Buckley 1980 [13]	2											32		**72**	26
Zaricznyj et al. 1980 [12]	12	40		4		**44**		16	12	8		8		**44**	
Mathur et al. 1981 [15]	5	17	4	2	1	24	14	12	11	46	2	6		**58**	
Rose 1981 [14]	11				1	5	4	27	15	9	11	11	2	**75**	5
Martin et al. 1987 [16]	22					0								**44**	33
Jamison and Lee 1989 (astroturf) [18]	15	16	2	1	1	21	5	3	4	15	13	18	7	**58**	
Jamison and Lee 1989 (grass) [18]	9	9	2	1	1	13	1	9	14	9	13	31	2	**77**	
Fuller 1990 [19]	10	18	2			20	9	5	9	5	17	24		**60**	
Powell and Barber-Foss 1999^a^ [21]	17		13		3	16	5	23				14	22	**59**	3
Finch et al. 2002^b^ [24]		31			4			28	12	19	30	31			
Junge et al. 2006 (men) [25]	22	6	9	3	3	16	8	14	3	3	8	22	0	**50**	
Junge et al. 2006 (women) [25]	**50**	25	0	0	0	25	13	13	0	0	0	0	0	13	
Dick et al. 2007 (game)^c^ [28]	25	10				21	7	15	3	3	10	18	3	**43**	4
Dick et al. 2007 (practice) [28]	8	2				8	16	15	2	8	27	17	12	**60**	7
Rishiraj et al. 2009 [29]	7	23			6	29	14	14			10	13	15	**53**	
Engebretsen et al. 2013 [31]	20	16	3		5	23	11	8	8	5	9	11	8	**47**	
Theilen et al. 2016 (men) [32]	27	19				19	4			13	28			**31**	9
Theilen et al. 2016 (women) [32]	**40**	14				14	0			16	12			28	18

Bold formatting indicates the highest values for the main body areas in each study

^a^Same data as Rauh et al. 2007 [27]

^b^Values represent percentages of injured players (i.e., not injuries) and do not add to 100% as some players sustained more than one injury

^c^Same data as Hootman et al. 2007 [26]

In total, 13 studies (59%) described the types of injury sustained by field hockey players [13–16, 18, 20, 21, 24, 25, 27–29, 31]. Table 5 presents the proportion (%) of injuries according to their type. Contusions and hematomas were the most common types of injury (ranging from 14% [31] to 64% [18] of all injuries), followed by abrasions and lacerations (5% [14] to 51% [15]), sprains (2% [18] to 37% [13]) and strains (0% [25] to 50% [28]). Concussions ranged from 0% [25] to 25% [25].

**Table 5:** Proportion (%) of field hockey injuries by injury type

Study	Sprains	Strains	Dislocation	Fracture	Abrasion, laceration	Contusion, hematoma	Swelling, blistering	Concussion	Tendinopathy	Other, unspecified
Clarke and Buckley 1980 [13]	**37**	21		7				4		32
Mathur et al. 1981 [15]	20^a^			6^b^	**51**		22	1		
Rose 1981 [14]	32	16	1	1	5	**33**		4	2	5
Martin et al. 1987 [16]	11				11	33				**44**
Jamison and Lee 1989 (astroturf) [18]	2	12			26	**49**	2	3		6
Jamison and Lee 1989 (grass) [18]	2	5			16	**64**	7	1		5
Cunningham and Cunningham 1996 [20]	15	19		2	22	**28**	3	4		9
Powell and Barber-Foss 1999^c^ [21]	26	20		6		**37**		3		8
Finch et al. 2002^d^ [24]	28	55	2	14	15	**80**		1	2	
Junge et al. 2006 (men) [25]	11	8	0	8	19	**42**		0	8	3
Junge et al. 2006 (women) [25]	13	0	0	0	25	**38**		25	0	0
Dick et al. 2007 (game)^e^ [28]	**24**	13		15	11	20		9	4	3
Dick et al. 2007 (practice)^e^ [28]	23	**50**		5			3^f^	3	7	8
Rishiraj 2009 [29]	10	**40**	1	1	8	17			24	
Engebretsen 2013 [31]	18	14	6	8	**21**	14			3	18

Bold formatting indicates the highest values for each study

^a^Sprains and strains reported together

^b^Fractures and dislocations reported together

^c^Same data as Rauh et al. 2007 [27]

^d^Values represent percentages of injured players (i.e. not injuries) and do not add to 100% as some players sustained more than one injury

^e^Same data as Hootman et al. 2007 [26]

^f^Reported as inflammation

#### Injury According to Mechanism and Player Position

Eight studies (36%) described injuries according to their mechanism [18–20, 25, 28, 29, 31, 32]. Table 6 presents the proportion (%) of injuries according to their mechanism. Non-contact injuries ranged from 12% [18] to 64% [28]. Contact with the ball (range: 2% [29] to 52% [32]) and stick (9% [29] to 27% [18]) were also common mechanisms, as was contact with another player (2% [19] to 45% [20]) or with the ground (9% [28] to 15% [20]).

**Table 6:** Proportion (%) of field hockey injuries by injury mechanism

First author, year	Ball contact	Stick contact	Player contact	Ground contact	Object contact	Unspecified contact	Noncontact	Unspecified
Jamison and Lee 1989 (astroturf) [18]	**32**	27	11	12			18	
Jamison and Lee 1989 (grass) [18]	**42**	23	9	14			12	
Fuller 1990 [19]	30	17	2	10			**41**	
Cunningham and Cunningham 1996 [20]			**45**	15			36	4
Junge et al. 2006 (men) [25]						**58**	36	6
Junge et al. 2006 (women) [25]						**75**	13	13
Dick et al. 2007 (game)^a^ [28]	**29**	18	14	9			28	2
Dick et al. 2007 (practice)^a^ [28]			5			26	**64**	5
Rishiraj et al. 2009 [29]	2	9	12	12	3^b^		**62**	
Engebretsen et al. 2013 [31]			8		**44** ^**c**^		41	7
Theilen et al. 2016 (men) [32]	**37**	25	23	15				
Theilen et al. 2016 (women) [32]	**52**	14	12	20				2

Bold formatting indicates the highest values for each study

^a^Same data as Hootman et al. 2007 [26]

^b^Contact with the goal

^c^Contact with unspecified moving or stagnant object

Three studies (14%) reported injuries according to the injured player’s position [19, 28, 29]. Goalkeepers sustained fewer injuries in all three studies that reported injuries by playing position (4% [19] to 19% [28]). Defenders sustained 16% [19] to 36% [29] of injuries, while midfielders and forwards sustained 22% [28] to 37% [19] (Table 7).

**Table 7:** Proportion (%) of field hockey injuries by player position

Study	Forwards	Midfielders	Defenders	Goalkeepers	Other, unknown
Rishiraj et al. 2009 [29]	32	22	**36**	10	
Fuller 1990 [19]	**37**	**37**	16	4	6
Dick et al. 2007 (game)^a^ [28]	22	**28**	24	19	7

Bold formatting indicates the highest values for each study

^a^Player position at time of injury. Same data as Hootman et al. 2007 [26]

## Discussion

To the best of our knowledge, the present study is the first systematic review to summarize the descriptive evidence of injuries sustained by field hockey players. We included only prospective studies to ensure we gathered the most reliable information available on the extent of injuries in field hockey in terms of rate and severity as well as injury characteristics according to body location, type, and mechanism of injury. To reduce and control field hockey injuries, as for all sports, we must first establish the extent of the injury problem [6]. The substantial heterogeneity between studies included in this review prevented conclusive findings on the extent of the rate and severity of injuries in field hockey (Tables 1, 2). Such heterogeneity may be caused by the different definitions and methods employed to record and report injuries and the different characteristics and levels of players studied.

This systematic review shows that, despite the long history of field hockey and its popularity worldwide, prospective studies focusing on overall field hockey injuries are still lacking. The majority of the studies investigated field hockey injuries together with injuries in other sports [11–13, 15–17, 20–27, 30, 31]. Within such studies, injury rates in field hockey were comparable to those in other team sports, such as basketball [23, 24, 26], netball [23, 24], lacrosse [26], and softball [21, 27]. The injury rate in field hockey can be considered low compared with football (soccer) [21, 25–27]. However, in major tournaments, the rate of time loss injuries in field hockey [32] can be considered higher than that in football (soccer) [4]. These findings confirm that the risk of sustaining an injury in field hockey should not be neglected.

Despite the considerable heterogeneity between studies, it is still possible to observe similar characteristics of injuries with regard to body location, type, and mechanism of injury. Most of the injuries described in the studies included in this review were to the lower limbs (Table 4), affecting mainly the knee and the ankle. This is in line with previous studies on team sports involving running and stepping maneuvers, such as football (soccer) [33] and lacrosse [34], and justifies a focus on preventive efforts in this body area. Interestingly, the majority of injuries sustained by women during major tournaments were to the head [25, 32]. A specific analysis of head injuries in collegiate women’s field hockey showed that 48% of these injuries occurred due to contact with an elevated ball [35]. Most (39%) of the concussions were due to direct contact with another player, and 25% were due to contact with an elevated ball [35].

Contusions and hematomas were common types of injury, as were abrasions and lacerations, which might be due to players’ contact with the ball, stick, and playing surface [2, 28]. A specific analysis of ball-contact injuries in 11 collegiate sports showed that injury rates were the highest in women’s softball, followed by women’s field hockey and men’s baseball [36]. In field hockey, the common activities associated with ball-contact injuries were defending, general play, and blocking shots [36]. To reduce the injury burden, the International Hockey Federation stated that goalkeepers must wear protective equipment comprising at least headgear, leg guards, and kickers [37]. Field players are recommended to use shin, ankle, and mouth protection [37], and other research suggested that the use of such equipment should be mandatory [2]. Accordingly, some national associations have updated their rules to make shin, ankle, and mouth protection obligatory [38, 39].

It is important to note that non-contact injuries are also a cause for concern in field hockey (Table 6). Although protective equipment has a fundamental role in injury prevention, it may not prevent most of the non-contact injuries. During the last decades, different studies have shown that it is possible to prevent injuries in team sports with structured exercise [40–44]. Yet, to our knowledge, evidence showing the implementation of such programs in field hockey is lacking. Nevertheless, exercise programs that have proven effective in preventing sports injury can be introduced as part of the regular training schedule of the field hockey team, especially programs focusing on the prevention of lower limb injuries [40–42]. While there is no structured exercise program for field hockey, stakeholders can also use open source resources for overall and specific injury prevention that are supported by the International Olympic Committee, such as exercise programs and guidelines on load management and youth athletic development [45–47].

### Future Recommendations

The present systematic review shows that studies have used different definitions and methods to record and classify injuries and their severity, and this prevents conclusive findings on the extent of the injury problem in field hockey. As establishing the extent of sports injury is considered the first step toward effective prevention [6], one of the main findings of this review is the recognition of the need for a consensus on the methodology of injury surveillance in field hockey. Consensus statements on the methodology of injury surveillance have been made available for a variety of sports [8, 48–54]. A consensus statement represents the result of a comprehensive collective analysis, evaluation, and opinion of a panel of experts regarding a specific subject (e.g., methodology of injury surveillance in field hockey) [55]. Consequently, consensus statements enable investigators from different settings to access and employ the same definitions and methods to collect and report injury data. Comparisons among different studies as well as data pooling for meta-analyses are then facilitated.

The common goal in field hockey is to promote players’ safety while maintaining the traditions of the sport [35]. Protecting the health of the athletes is also a priority of the International Olympic Committee [56], and resources for injury prevention have been made available for the public in general [45–47]. The field hockey community would benefit from studies investigating the implementation of such resources and from strategies that have been proven to be effective in other sports [40–44]. Until there is consensus on the methodology of injury surveillance in field hockey, investigators may use consensus from other team sports in future studies as an example [8, 52, 53]. Based on the gaps identified in the studies included in this review, the authors also suggest that future studies adhere to the reporting guidelines from the Enhancing the Quality and Transparency of Health Research (EQUATOR) Network. The EQUATOR Network provides comprehensive documentation on what information needs to be reported in scientific manuscripts depending on the study design [57]. By following an appropriate guideline such as that of the EQUATOR Network, future investigators will facilitate assessment of the generalizability, strengths, and limitations of studies on field hockey injuries.

### Limitations

Electronic searches were conducted in four databases that were considered relevant for this systematic review. This does not rule out the possibility of eligible articles published in journals that were not indexed in any of these databases. To minimize this limitation, we screened the references of the full texts assessed for eligibility and included additional studies that were not identified in the database search. In addition, this systematic review included only scientific manuscripts published in English, although studies on field hockey injuries have been published in other languages. These were not included because the authors were unable to translate the papers accurately enough to extract their data.

## Conclusion

The present systematic review shows that, despite the long history and the popularity of field hockey worldwide, few prospective studies have investigated the overall injury problem in field hockey. Most of the information on field hockey injuries registered prospectively comes from studies conducted in multi-sport settings. The range of definitions, methods, and reporting employed by studies prevents conclusive findings on the rate and severity of injuries in field hockey. To facilitate the development of evidence-based strategies for injury prevention, field hockey may benefit from a consensus on the methodology of injury surveillance. While no specific consensus is available for field hockey, future studies may use widely accepted consensus from other sports, such as football (soccer). In addition, future studies on field hockey injuries are encouraged to adhere to the reporting guidelines from the EQUATOR Network.

Despite the considerable heterogeneity, it is clear that most of the injuries sustained by field hockey players affect the lower limbs, justifying efforts to develop preventive strategies for this body area. Contact injuries, such as contusions/hematomas, and abrasions, are frequent, and the use of protective equipment for the ankle, shin, hand, mouth, and eye/face has been recommended. Nevertheless, non-contact injuries are also common in field hockey, and most of these may not be prevented by protective gear. To reduce the burden of injuries, field hockey stakeholders may implement exercise-based injury-prevention programs and guidelines on load management and youth athletic development that have been supported by the International Olympic Committee.

## Electronic supplementary material

Below is the link to the electronic supplementary material.

Supplementary material 1 (DOCX 512 kb)
