# Supplementary material for: Injuries in Field Hockey Players: A Systematic Review
Source: Sports Med. 2018 Jan 3;48(4):849–66. doi: 10.1007/s40279-017-0839-3 (PMC5856874; doi:10.1007/s40279-017-0839-3)
Supplement: Supplementary file 1 — Supplementary material 1 (DOCX 512 kb) [file 40279_2017_839_MOESM1_ESM.docx]

# Injuries in field hockey players: a systematic review

Journal: Sports Medicine

Saulo Delfino Barboza^1^, Corey Joseph^2^, Joske Nauta^1^, Willem van Mechelen^1,3,4,5^, Evert Verhagen^1,2,3^

^1^Amsterdam Collaboration on Health and Safety in Sports, Department of Public and Occupational Health, Amsterdam Public Health research institute, VU University Medical Center Amsterdam, Amsterdam, The Netherlands.

^2^Australian Collaboration for Research into Injury in Sport and its Prevention, Federation University Australia, Ballarat, VIC, Australia.

^3^School of Human Movement and Nutrition Sciences, Faculty of Health and Behavioural Sciences, University of Queensland, Brisbane, QLD, Australia.

^4^ Division of Exercise Science and Sports Medicine (ESSM), Department of Human Biology, Faculty of Health Sciences, University of Cape Town, Cape Town, South Africa.

^5^School of Public Health, Physiotherapy and Population Sciences, University College Dublin, Dublin, Ireland.

**Corresponding author:** Evert Verhagen ([e.verhagen@vumc.nl)](mailto:e.verhagen@vumc.nl))

****Electronic Supplementary Material Appendix S1.**** **Literature search strategy and searched databases.**

## Search strategy for PubMed

## Part A: search for studies about injury

1. injur*
2. traum*
3. risk*
4. overuse
5. overload
6. acute
7. odds
8. incidence
9. prevalence
10. hazard
11. OR/1-10

## Part B: search for studies about field hockey

1. field
2. hockey
3. ice
4. 12 AND 13 OR 12 NOT 14

## Part C: search for prospective and retrospective studies

1. prosp*
2. retrosp*
3. case*
4. OR/16-18

## Full search in PubMed (386 records)

1. 11 AND 15 AND 19

#

# Search strategy for Exerpta Medica Database (EMBASE)

## Part A: search for studies about injury

1. injur*
2. traum*
3. risk*
4. overuse
5. overload
6. acute
7. odds
8. incidence
9. prevalence
10. hazard
11. OR/21-30

## Part B: search for studies about field hockey

1. field
2. hockey
3. ice
4. 32 AND 33 OR 32 NOT 34

## Part C: search for prospective and retrospective studies

1. prosp*
2. retrosp*
3. case*
4. OR/36-38

## Full search in EMBASE (146 records)

1. [embase]/lim
2. [medline]/lim
3. 31 AND 35 AND 39 AND 40 NOT 41

# Search strategy for SPORTDiscus [via EBSCO]

## Part A: search for studies about injury

1. injur*
2. traum*
3. risk*
4. overuse
5. overload
6. acute
7. odds
8. incidence
9. prevalence
10. hazard
11. OR/43-52

## Part B: search for studies about field hockey

1. field
2. hockey
3. ice
4. 54 AND 55 OR 54 NOT 56

## Part C: search for prospective and retrospective studies

1. prosp*
2. retrosp*
3. case*
4. OR/58-60

## Full search in SPORTDiscus (168 records)

1. 53 AND 57 AND 61 [limit: academic journals]

# Search strategy for Center on Health Sciences Information (CINAHL) [via EBSCO]

## Part A: search for studies about injury

1. injur*
2. traum*
3. risk*
4. overuse
5. overload
6. acute
7. odds
8. incidence
9. prevalence
10. hazard
11. OR/63-72

## Part B: search for studies about field hockey

1. field
2. hockey
3. ice
4. 74 AND 75 OR 74 NOT 76

## Part C: search for prospective and retrospective studies

1. prosp*
2. retrosp*
3. case*
4. OR/78-80

## Full search in CINAHL (66 records)

1. 73 AND 77 AND 81 [limit: academic journals]
